# A SMART approach to reducing paroxysmal atrial fibrillation symptoms: Results from a pilot randomized controlled trial

**DOI:** 10.1016/j.hroo.2021.06.003

**Published:** 2021-06-22

**Authors:** Michelle L. Dossett, Emma W. Needles, Zachary Donahue, Gillian Gadenne, Eric A. Macklin, Jeremy N. Ruskin, John W. Denninger

**Affiliations:** ∗ Department of Medicine, University of California Davis, Sacramento, California; † Department of Medicine, Massachusetts General Hospital, Boston, Massachusetts; ‡ Benson-Henry Institute for Mind Body Medicine, Massachusetts General Hospital, Boston, Massachusetts; § Biostatistics Center, Massachusetts General Hospital, Boston, Massachusetts; ‖ Department of Psychiatry, Massachusetts General Hospital, Boston, Massachusetts

**Keywords:** Meditation, Paroxysmal atrial fibrillation, Psychological resilience, Psychological stress, Quality of life

## Abstract

**Background:**

Stress and negative emotions contribute to atrial fibrillation (AF). Mind-body practices decrease stress and negative emotions and may reduce AF episodes and improve quality of life for patients with AF.

**Objective:**

We examined the effects of a multimodal mind-body program, the SMART Program, on AF-related quality of life in patients with paroxysmal AF (PAF).

**Methods:**

In this randomized, waitlist-controlled pilot trial, 18 subjects with PAF participated in an 8-week SMART Program delivered online immediately or 3 months later. Validated measures were completed at baseline and at 3 and 6 months (waitlist group only).

**Results:**

Comparing pre- vs post-program scores among all 18 participants, subjects reported improvement in AF-related quality of life (Cohen’s d = 0.75, *P* = .005) and depression (d = 0.50, *P* = .05) but not anxiety (d = 0.35, *P* = .16). Subjects also reported improvements in AF symptom severity (*P* = .026), distress (*P* = .014), positive affect (*P* = .003), and ability to cope with stress (*P* = .001). Compared to waitlist control subjects, those in the immediate group reported improvement in positive affect (d = 1.20, *P* = .021) and coping with stress (d = 1.36, *P* = .011) after participating in the program.

**Conclusion:**

The SMART Program, delivered virtually, may enhance positive emotions and coping with stress as well as decrease negative emotions and AF symptoms. These results warrant a larger trial to better understand the potential benefits of such programs for patients with PAF.

**Key Findings**

▪ In this pilot randomized, waitlist-controlled trial, an 8-week multimodal Stress Management and Resiliency Training Program (SMART Program) significantly improved AF-related quality of life in patients with symptomatic paroxysmal atrial fibrillation.
▪ Program participants also noted improvements in coping with stress, positive affect, and depression.
▪ Telehealth delivery of group-based stress reduction programs is feasible for older adults and may facilitate their ability to access and learn these skills.

## Introduction

Atrial fibrillation (AF) is the most common cardiac arrhythmia, with a lifetime risk of 1 in 6 individuals, and its incidence increases with age.^1^^,^^2^ AF is associated with increased risk of stroke as well as increased risk of cognitive impairment and dementia, regardless of stroke history.^3^ Approximately one-third of patients with AF have depression, anxiety, or both.^4^ Among patients with paroxysmal atrial fibrillation (PAF), quality of life as measured by the SF-36 (36-Item Short Form Survey) is as low as in patients with significant structural heart disease (eg, post myocardial infarction or heart failure).^5^ Large epidemiologic studies have demonstrated an association between chronic stress and development of AF.^6^^,^^7^ Moreover, in patients with PAF, acute stress and negative emotions (eg, sadness, anger, anxiety, and impatience) are associated with 2- to 5-fold higher odds of an AF episode on the following day, whereas happiness is associated with an 85% lower odds of AF on the following day.^8^ Moreover, the effect of acute stress and negative emotions on triggering an AF episode is significantly attenuated in individuals taking beta blockers (suggesting that this class of medications may be attenuating stress-induced adrenergic signaling).^9^

Mind-body techniques such as meditation and yoga decrease the physiologic and psychologic stress response, resulting in decreased catecholamine release, reduction in negative emotions, and increased positive affect.^10^, ^11^, ^12^, ^13^, ^14^ The physiologic effects of these practices may be particularly relevant for modulating the autonomic nervous system and decreasing the frequency of AF episodes.^15^ Indeed, data suggest that mind-body interventions such as yoga and meditation may reduce stress, improve quality of life, and possibly reduce AF frequency in patients with PAF. A single arm, pre-post study of a 12-week yoga and relaxation program for 60 patients with PAF found a reduction in both symptomatic and asymptomatic AF episodes, symptomatic non-AF episodes, and anxiety and depression in participants after completing the intervention.^16^ A 12-week randomized controlled trial of yoga for patients with PAF found improved quality of life and decreased blood pressure and heart rate.^17^ A randomized controlled trial of a 9-week mindfulness and cognitive behavioral therapy program vs usual care (n = 104) found improved quality of life and reduced depression in the intervention group a year later.^18^ A pilot study of Vipassana meditation in 25 patients with congestive heart failure and implanted cardiac defibrillators followed for an average of 6.5 years found less sustained AF and VT in the meditation group compared to a usual care control group; however, the meditation group was also significantly younger at baseline.^19^

A number of programs have been developed to teach mind-body skills to patients in a clinical setting. One such program with which we have extensive experience, and which has been shown to improve quality of life and symptoms in multiple patient populations, including patients with coronary artery disease, is the Stress Management and Resiliency Training: Relaxation Response Resiliency Program (SMART-3RP or SMART Program for short).^20^, ^21^, ^22^, ^23^, ^24^, ^25^, ^26^ Based upon our clinical experience with this program in this patient population and the above literature, we conducted a pilot clinical trial of the SMART Program for patients with PAF to determine whether it could improve quality of life for patients with PAF and reduce anxiety and depression. To improve access and facilitate subject recruitment, program sessions were held online using a secure telehealth platform.

## Methods

### Trial design

This study was a randomized, parallel-assignment pilot study to investigate the effects of the Stress Management and Resiliency Training (SMART) Program^20^ on quality of life in patients with symptomatic PAF. The study was approved by the Partners Human Research Committee/IRB and conducted according to the principles of the Declaration of Helsinki. All participants provided written informed consent. The trial is registered on ClinicalTrials.gov (NCT03450993).

### Participants

Patients were eligible if they were 18–90 years of age, were able to understand written and spoken English, had documented recurrent symptomatic PAF, were on stable medical therapy for their PAF, and were willing to continue on the same treatment regimen while participating in the study. Individuals were excluded if they had a current daily practice of yoga, meditation, guided imagery, or other similar mind-body techniques; were unwilling or unable to participate in an intervention delivered via video conferencing; unable to come to the research center to complete outcome measures; had end stage renal disease or heart failure or a severe unstable medical or psychiatric disease; or were unable to complete the protocol due to cognitive reasons. Patients on psychoactive medications had their eligibility assessed on a case-by-case basis depending on the stability of their medication regimen.

### Intervention

The SMART Program is a manualized psychoeducational program that teaches participants a variety of different skills to more effectively cope with stress and reduce its negative health effects.^20^ The program includes training in mind-body skills (meditation techniques, mini relaxations, mindfulness, and yoga), stress awareness tools, healthy lifestyle behaviors (sleep, exercise, nutrition, and social support), and cognitive reappraisal and adaptive coping skills (adapted from cognitive behavioral therapy, acceptance and commitment therapy, and positive psychology). Sessions are held weekly for 1.5 hours for 8 weeks and include didactic components, experiential exercises, and small group activities. As part of the program, subjects received a workbook and recorded guided meditations and were encouraged to practice for 10-20 minutes a day and to complete a daily log of their practice. Although many of the tools taught in the program are designed to be integrated as part of a daily practice/lifestyle, participants were particularly encouraged to use one of the mini relaxations taught in the program if they developed cardiac symptoms (e.g., palpitations) as a way to both decrease autonomic dysregulation and any attendant anxiety.

All SMART group sessions were conducted online using a hospital-approved, secure video telehealth platform (Vidyo, https://www.vidyo.com/) that allows for encrypted audio and video transmission and were led by a single physician (MLD) experienced in running these groups. Sessions were held in the evening hours during the work week. A study research coordinator worked with each participant prior to the first session of the SMART Program to assist them in downloading the required software and to help them become comfortable connecting with and using the virtual environment.

### Outcomes

The primary outcomes were changes in AF-related quality of life (Atrial Fibrillation Effect on Quality of Life Questionnaire – AFEQT^27^), anxiety (GAD-7^28^), and depression (PHQ-8^29^) over approximately 3 months from before to after the program across all participants. Given the pilot nature of this study, we also asked subjects to complete additional questionnaires to better understand the psychosocial effects of the SMART Program in this patient population and the mechanisms underlying those effects. These questionnaires included the Atrial Fibrillation Symptom Severity and Burden Questionnaire,^30^ Distress Scale (0–10 scale), the Penn State Worry Questionnaire (PSWQ)^31^, positive affect portion of the Positive and Negative Affect Scale (PANAS^32^), Happiness Scale^8^ (0–6 scale), the Measure of Current Status – A (MOCS-A,^33^ a measure of stress reactivity/coping), Perceived Stress Scale-10 (PSS-10^34^), the Current Experiences Scale (modified from the Post-Traumatic Growth Inventory,^35^ a measure of resilience), and the Cognitive and Affective Mindfulness Scale-Revised (CAMS-R^36^). Subjects completed postprogram measures within 3–5 weeks of completing the SMART Program. Subjects in the immediate group completed outcome measures twice (pre- and post-intervention, at baseline and 3 months). Subjects in the waitlist control group completed measures 3 times (at baseline [3 months preintervention], 3 months [immediately preintervention], and 6 months [postintervention]).

### Randomization and Blinding

The study statistician generated the randomization allocation table and uploaded it into the study REDCap^37^ database. No other study personnel had access to the randomization sequence. Subjects were randomized by the study research coordinator after all baseline data were collected. Subjects were randomized 1:1 to receive the SMART Program either immediately (n = 9) or 3 months later (n = 9, waitlist control group). A diagram illustrating time points for outcome assessments for both groups is shown in Supplemental Appendix 1. Following randomization, study participants and the research team knew individuals’ group assignments; however, initial data analysis was performed blinded.

### Statistical analysis

All analyses were conducted in SPSS v24 (IBM Corp, Armonk, NY). Descriptive statistics were used to assess participant demographics and assess the normality of questionnaire data. Although most of the data were approximately normally distributed, a few measures were not; however, nonparametric tests yielded equivalent inference to the parametric tests for these measures, and thus all reported values are from parametric tests. For the several instances in which a question was skipped by a study participant, data were assumed to be missing at random and missing data were imputed by calculating the average score for a measure for the answered items. To compare pre- vs post-intervention scores for all 18 subjects, paired samples *t* tests were used (using the second and third assessments for the waitlist control group). Independent samples *t* tests were used to compare changes in scores for the immediate vs waitlist control group (using the first and second assessments for the control group). As this small study was intended primarily as a hypothesis-generating pilot study, *P* values were not corrected for multiple comparisons.

## Results

**Figure 1:**
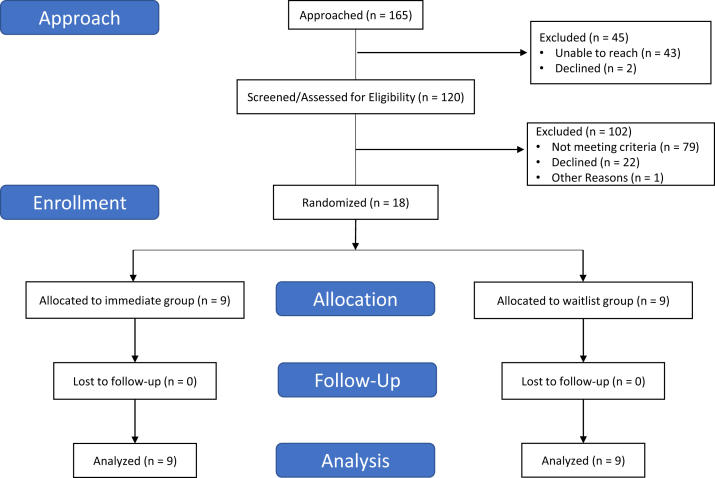
CONSORT flow diagram. The most common reasons potential subjects were ineligible were that they did not have paroxysmal atrial fibrillation (AF) (n = 26, either continuous AF or no evidence of AF at all), that they had no recent AF episode (>1 year, n = 14), or that they were unable or unwilling to travel to the study site (n = 10). Some individuals could not make the time to participate in the intervention if randomized to it (n = 6), had a daily meditation practice already (n = 5), or did not have computer access (n = 5). One subject was eligible but not randomized because the study filled before they called back to schedule an appointment.

A total of 18 individuals with PAF enrolled in the study (Figure 1). Participant demographics are presented in Table 1. The mean age was 66 years, 14 (78%) were women, and the majority were college graduates (72%). The immediate and waitlist control groups were similar in demographics, but subjects in the waitlist control group were taking more cardiac medications, particularly antiarrhythmics, and had a lower baseline quality-of-life scores on the AFEQT. Participants attended 7.2 sessions on average (range 6–8 sessions).

**Table 1:** Participant demographics

	Participant group		
	Immediate (n = 9)	Waitlist (n = 9)	Total (n = 18)
Age (mean ± SD), years	66 ± 3.3	66 ± 7.2	66 ± 5.5
Female sex	6 (67)	8 (89)	14 (78)
Race			
White	9 (100)	9 (100)	18 (100)
Ethnicity			
Hispanic or Latino	0 (0)	0 (0)	0 (0)
Education			
Diploma program	2 (22)	3 (33)	5 (28)
College graduate	7 (78)	6 (67)	13 (72)
Marital status			
Married	6 (67)	5 (56)	11 (61)
Divorced/separated	3 (33)	2 (22)	5 (27)
Widowed	0 (0)	1 (11)	1 (6)
Never married	0 (0)	1 (11)	1 (6)
Employment			
Employed for wages	6 (67)	5 (56)	11 (61)
Self-employed	1 (11)	0 (0)	1 (6)
Retired	2 (22)	4 (44)	6 (33)
Medications			
Beta blocker	5 (56)	5 (56)	10 (56)
Non-DHP CCB	0 (0)	2 (22)	2 (11)
Antiarrhythmics	3 (33)	8 (89)	11 (61)
Antihypertensives	2 (22)	6 (67)	8 (44)
Anticoagulants	6 (67)	8 (89)	14 (78)
Aspirin	3 (33)	2 (22)	5 (28)
Statin	1 (11)	5 (56)	6 (33)
Diuretic	0 (0)	2 (22)	2 (11)

Results are n (%) unless indicated.

Non-DHP CCB = non-dihydropyridine calcium channel blocker.

Examination of pre-post program changes for all 18 subjects demonstrated significant improvements in AF-related quality of life (*P* = .005), AF symptom severity (*P* = .026), distress (*P* = .014), positive affect (*P* = .003), and stress reactivity/coping (*P* = .001) with moderate-to-large effect sizes (Table 2). Nonsignificant changes with moderate effect sizes were also seen for improvements in depression, anxiety, happiness, perceived stress, and resilience.

**Table 2:** Pre-post intervention changes (n = 18 participants)

	Pre mean (SD)	Post mean (SD)	*P* value	Cohen’s d
AF quality of life	74.7 (18.6)	85.8 (14.9)	.005	0.75
Depression	4.8 (4.6)	2.9 (4.0)	.05	0.50
Anxiety	7.7 (5.3)	5.4 (6.2)	.155	0.35
AF severity	12.6 (6.0)	9.2 (5.9)	.026	0.59
AF burden	3.4 (1.8)	3.1 (1.6)	.408	0.20
Distress	5.1 (2.5)	3.3 (2.6)	.014	0.64
Worry	50.8 (14.6)	47.1 (13.9)	.214	0.30
Positive affect	34.4 (6.0)	38.3 (6.5)	.003	0.81
Happiness	3.8 (1.0)	4.1 (2.6)	.055	0.49
Stress reactivity/coping	23.4 (11.3)	31.7 (8.0)	.001	0.97
Perceived stress	17.6 (5.7)	14.9 (6.3)	.06	0.47
Resilience	90.8 (15.2)	94.9 (15.7)	.146	0.36
Mindfulness	31.1 (4.7)	32.2 (3.6)	.251	0.28

AF = atrial fibrillation.

Comparison of the immediate intervention group that received the SMART Program to the waitlist control group before they received the SMART Program (9 vs 9 subjects) demonstrated significant improvements in stress reactivity/coping (*P* = .01) and positive affect (*P* = .02) with large effect sizes (Table 3). Nonsignificant changes with large effect sizes were also seen for improvements in AF-related quality of life, depression, and happiness.

**Table 3:** Intervention vs waitlist comparisons (n = 18, 9 vs 9)

	Group	Pre mean (SD)	Post mean (SD)	*P* value	Cohen’s d
AF quality of life	I	84.1 (10.6)	91.3 (4.8)	.086	0.86
	W	66.5 (20.2)	65.4 (20.6)		
Depression	I	5.1 (4.3)	2.0 (1.4)	.073	0.9
	W	4.0 (3.8)	4.4 (5.2)		
Anxiety	I	7.8 (5.1)	5.0 (3.6)	.617	0.24
	W	9.0 (5.9)	7.6 (5.7)		
AF severity	I	10.9 (3.8)	8.1 (4.7)	.202	0.64
	W	14.1 (7.2)	14.1 (7.3)		
AF burden	I	3.2 (2.2)	3.6 (1.4)	1	0
	W	3.3 (1.9)	3.7 (1.3)		
Distress	I	5.2 (1.9)	3.6 (1.9)	.203	0.63
	W	4.7 (2.4)	4.9 (3.1)		
Worry	I	49.6 (15.8)	45.6 (12.1)	.792	0.13
	W	54.6 (17.1)	52.0 (14.2)		
Positive affect	I	32.4 (5.8)	37.4 (7.1)	.021	1.2
	W	37.0 (7.1)	36.3 (5.7)		
Happiness	I	3.9 (0.8)	4.3 (0.5)	.089	0.85
	W	3.9 (0.9)	3.7 (1.2)		
Stress reactivity / coping	I	23.0 (10.3)	30.8 (4.4)	.011	1.36
	W	25.7 (12.7)	23.9 (12.8)		
Perceived stress	I	17.9 (4.8)	15.4 (3.4)	.587	0.26
	W	18.2 (6.1)	17.2 (6.8)		
Resilience	I	87.7 (17.2)	90.7 (16.1)	.57	0.27
	W	15.6 (94.0)	94.0 (13.0)		
Mindfulness	I	30.3 (5.7)	31.3 (3.8)	1	0
	W	30.9 (6.0)	31.9 (3.5)		

I = intervention; W = waitlist.

## Discussion

In this virtually delivered pilot study of the SMART Program for patients with symptomatic PAF, subjects noted significantly improved AF-related quality of life and reduced depressive symptoms and no significant change in anxiety. The largest effects of the program were improvements in stress reactivity/coping and increases in positive affect.

Although subjects in the waitlist control group were taking more antiarrhythmics and other medications and had a lower quality of life scores on the AFEQT compared to the immediate group, potentially confounding the comparison between the immediate and waitlist control groups, subjects in the waitlist group also noted a significant improvement in their AFEQT score after completing the SMART Program (mean 80.7, standard deviation 19.8, *P* = .037).

Programs teaching mind-body skills have been shown to decrease anxiety and depression in a variety of patient populations.^12^^,^^13^ Anxiety and depression commonly co-occur in patients with PAF, and these negative emotions not only increase stress and decrease quality of life, but may also increase autonomic dysregulation,^7^^,^^15^ increasing the potential for AF episodes.^4^^,^^8^^,^^15^ Notably, our small sample was not particularly anxious or depressed, which may explain the lack of significant changes on these measures in our study. Nonetheless, we saw moderate-to-large effect sizes for decreases in depressive symptoms. Perhaps even more importantly, we found significant improvements in positive affect and moderate-to-large effect sizes for improvements in happiness following participation in the SMART Program. Positive emotions have been shown to significantly reduce the likelihood of AF episodes.^6^^,^^8^

Consistent with the set of skills taught in the SMART Program, and the changes in positive and negative affect observed, participants reported significant improvements in stress reactivity and coping after completing the program. We also observed nonsignificant, moderate effect size reductions in perceived stress and reductions in distress.

Prior studies of mind-body interventions for patients with AF or significant structural heart disease have shown decreases in arrhythmia frequency.^16^^,^^19^ Although we did not measure this objectively, subjects did report decreased AF symptom severity (frequency) on a standardized questionnaire. No difference in burden of AF symptoms was reported; however, the latter assessed a 6-month period, which was too long for this study. If mind-body approaches do indeed decrease frequency of AF, our results suggest that the primary mechanism may be improving individuals’ abilities to respond to stress in a healthy manner and increasing positive affect. Significantly, these results may be achievable even with modest increases in mindfulness.

A notable feature of this study is the virtual delivery of the program. This study took place prior to the COVID-19 pandemic when virtual delivery of group-based programs and telehealth visits were relatively uncommon. Nonetheless, the technology was not a significant barrier to subject participation (only 5 of the potential subjects we screened did not have computer/technology access). Participants appreciated not having to travel to the hospital every week to participate in the program, and fewer than 1 session of 8 was missed on average. A prior pilot that we attempted with in-person groups was difficult to enroll, as weekly travel to the hospital was a significant barrier to participation. Nonetheless, several participants had low computer literacy, and their participation was enabled only through one-on-one training with the study coordinator. Thus, even individuals with limited computer literacy can be taught how to engage with, and benefit from, online programs.

Limitations of our work include the small sample size, that all groups were run by a single clinician, chance confounding between random treatment assignment and use of antiarrhythmics and baseline quality of life scores, a demographically homogeneous sample, and that there was no long-term follow-up. Nonetheless, the diversity of mind-body programs that have demonstrated improved quality of life for patients with PAF suggests that this effect may be real and that further research to understand the mechanisms, as well as larger trials, are warranted. Moreover, even 8-week mind-body skills–based interventions have been reported to have significant benefits out to at least 1 year.^18^^,^^38^ Our patient population was predominantly female and entirely white, and most individuals had a college degree, limiting generalization to other populations. Finally, we were unable to collect objective data on AF burden in study participants. Nonetheless, as mind-body interventions have demonstrated decreased health care utilization and costs in other conditions,^21^^,^^39^ incorporation of these tools into clinical care for patients with PAF may make sense not only from a quality-of-life standpoint but also from a health economics standpoint, particularly given the high costs of AF and related sequelae.^40^

## Conclusion

We found that an 8-week mind-body program, known as the SMART Program, delivered in a group-based, online format, improves AF-related quality of life, stress reactivity/coping, and happiness in patients with PAF. This program may also decrease negative emotions and AF severity. Given these results, larger trials of the SMART program in this patient population are warranted. If benefit is confirmed, the ability to deliver this intervention virtually will facilitate dissemination of these tools to patients as part of clinical care.

## Acknowledgments

We thank the following individuals for their assistance with this study and/or a previous pilot: Jennifer Galvin, Seyed Hosseini, Ihsan Kaadan, Alexander Nguyen, Ayman Shaqdan, Jeena Vaid, and Mohamed Youniss.

## Funding Sources

Funding for this study was provided by a grant from the Osher Center for Integrative Medicine at Harvard Medical School. Funding for preliminary work came from the Harold Grinspoon Charitable Foundation. MLD was funded by K23AT009218 from the National Center for Complementary and Integrative Health (NCCIH) at NIH. The project described was also supported by Grant Numbers 1UL1TR002541-01 and 1UL1TR001102 from NIH. The content is solely the responsibility of the authors and does not necessarily represent the official views of the National Institutes of Health.

## Disclosures

Dr Dossett is an author for UpToDate. Dr Macklin received grant support through his institution from Alector, Amylyx Pharmaceuticals, Biohaven Pharmaceuticals, Clene Nanomedicine, GlaxoSmithKline, Mitsubishi Tanabe Pharma America, Prilenia Therapeutics, and UCB Ra Pharma; served as a Steering Committee Member for Biogen and Stoparkinson Healthcare Systems; served as a DSMB member for Novartis Pharmaceuticals and Shire Human Genetic Therapies; and served on advisory boards for Bial Biotech, Cortexyme, Enterin, and Partners Therapeutics. Dr Ruskin has served as a consultant for Advanced Medical Education, InCardia, and Janseen; and has an equity interest in Ablacor, Celero Systems, Element Science, Infobionic, LuxMed, and NewPace. Dr Denninger reports research support for investigator-initiated studies from Basis/Intel and Onyx/Amgen.

## Authorship

All authors attest they meet the current ICMJE criteria for authorship.

## Patient Consent

All participants provided written informed consent.

## Ethics Statement

The study was approved by the Partners Human Research Committee/IRB and conducted according to the principles of the Declaration of Helsinki.

## References

[1] Lloyd-Jones D.M., Wang T.J., Leip E.P. (2004). Lifetime risk for development of atrial fibrillation: the Framingham Heart Study. Circulation.

[2] Fuster V., Rydén L.E., Cannom D.S. (2011). 2011 ACCF/AHA/HRS focused updates incorporated into the ACC/AHA/ESC 2006 Guidelines for the management of patients with atrial fibrillation: a report of the American College of Cardiology Foundation/American Heart Association Task Force on Practice Guidelines developed in partnership with the European Society of Cardiology and in collaboration with the European Heart Rhythm Association and the Heart Rhythm Society. J Am Coll Cardiol.

[3] Kalantarian S., Stern T.A., Mansour M., Ruskin J.N. (2013). Cognitive impairment associated with atrial fibrillation: a meta-analysis. Ann Intern Med.

[4] Thrall G., Lip G.Y.H., Carroll D., Lane D. (2007). Depression, anxiety, and quality of life in patients with atrial fibrillation. Chest.

[5] Dorian P., Jung W., Newman D. (2000). The impairment of health-related quality of life in patients with intermittent atrial fibrillation: implications for the assessment of investigational therapy. J Am Coll Cardiol.

[6] Lampert R. (2016). Behavioral influences on cardiac arrhythmias. Trends Cardiovasc Med.

[7] Kivimäki M., Steptoe A. (2018). Effects of stress on the development and progression of cardiovascular disease. Nat Rev Cardiol.

[8] Lampert R., Jamner L., Burg M. (2014). Triggering of symptomatic atrial fibrillation by negative emotion. J Am Coll Cardiol.

[9] Lampert R., Burg M.M., Jamner L.D. (2019). Effect of β-blockers on triggering of symptomatic atrial fibrillation by anger or stress. Heart Rhythm.

[10] Dusek J.A., Benson H. (2009). Mind-body medicine: a model of the comparative clinical impact of the acute stress and relaxation responses. Minn Med.

[11] Curiati J.A., Bocchi E., Freire J.O. (2005). Meditation reduces sympathetic activation and improves the quality of life in elderly patients with optimally treated heart failure: a prospective randomized study. J Altern Complement Med N Y N.

[12] Goyal M., Singh S., Sibinga E.M.S. (2014). Meditation programs for psychological stress and well-being: a systematic review and meta-analysis. JAMA Intern Med.

[13] Hempel S, Taylor SL, Marshall NJ, et al. Evidence Map of Mindfulness [Internet]. Washington (DC): Department of Veterans Affairs (US); 2014. [cited 2019 Mar 25], http://www.ncbi.nlm.nih.gov/books/NBK268640/. 25577939

[14] Hilton L.G., Marshall N.J., Motala A. (2019). Mindfulness meditation for workplace wellness: an evidence map. Work Read Mass.

[15] Bashir M.U., Bhagra A., Kapa S., McLeod C.J. (2019). Modulation of the autonomic nervous system through mind and body practices as a treatment for atrial fibrillation. Rev Cardiovasc Med.

[16] Lakkireddy D., Atkins D., Pillarisetti J. (2013). Effect of yoga on arrhythmia burden, anxiety, depression, and quality of life in paroxysmal atrial fibrillation: the YOGA My Heart Study. J Am Coll Cardiol.

[17] Wahlstrom M., Rydell Karlsson M., Medin J., Frykman V. (2017). Effects of yoga in patients with paroxysmal atrial fibrillation - a randomized controlled study. Eur J Cardiovasc Nurs.

[18] Malm D., Fridlund B., Ekblad H., Karlström P., Hag E., Pakpour A.H. (2018). Effects of brief mindfulness-based cognitive behavioural therapy on health-related quality of life and sense of coherence in atrial fibrillation patients. Eur J Cardiovasc Nurs.

[19] Aditee D., Pankaj M., Neil B., Nayereh P., Dali F., N Srivatsa U. (2020). Meditation for improved clinical outcomes in patients with implantable defibrillators for heart failure - pilot study. J Atr Fibrillation.

[20] Park E.R., Traeger L., Vranceanu A.-M. (2013). The development of a patient-centered program based on the relaxation response: the Relaxation Response Resiliency Program (3RP). Psychosomatics.

[21] Stahl J.E., Dossett M.L., LaJoie A.S. (2015). Relaxation response and resiliency training and its effect on healthcare resource utilization. PloS One.

[22] Vranceanu A.-M., Gonzalez A., Niles H. (2014). Exploring the effectiveness of a modified comprehensive mind-body intervention for medical and psychologic symptom relief. Psychosomatics.

[23] Zeng W., Stason W.B., Fournier S. (2013). Benefits and costs of intensive lifestyle modification programs for symptomatic coronary disease in Medicare beneficiaries. Am Heart J.

[24] Kuo B., Bhasin M., Jacquart J. (2015). Genomic and clinical effects associated with a relaxation response mind-body intervention in patients with irritable bowel syndrome and inflammatory bowel disease. PloS One.

[25] Park E.R., Perez G.K., Millstein R.A. (2020). A virtual resiliency intervention promoting resiliency for parents of children with learning and attentional disabilities: a randomized pilot trial. Matern Child Health J.

[26] Kuhlthau K.A., Luberto C.M., Traeger L. (2020). A virtual resiliency intervention for parents of children with autism: a randomized pilot trial. J Autism Dev Disord.

[27] Spertus J., Dorian P., Bubien R. (2011). Development and validation of the Atrial Fibrillation Effect on QualiTy-of-Life (AFEQT) Questionnaire in patients with atrial fibrillation. Circ Arrhythm Electrophysiol.

[28] Spitzer R.L., Kroenke K., Williams J.B.W., Löwe B. (2006). A brief measure for assessing generalized anxiety disorder: the GAD-7. Arch Intern Med.

[29] Kroenke K., Strine T.W., Spitzer R.L., Williams J.B.W., Berry J.T., Mokdad A.H. (2009). The PHQ-8 as a measure of current depression in the general population. J Affect Disord.

[30] Koci F., Forbes P., Mansour M.C. (2014). New classification scheme for atrial fibrillation symptom severity and burden. Am J Cardiol.

[31] Meyer T.J., Miller M.L., Metzger R.L., Borkovec T.D. (1990). Development and validation of the Penn State Worry Questionnaire. Behav Res Ther.

[32] Watson D., Clark L.A., Tellegen A. (1988). Development and validation of brief measures of positive and negative affect: the PANAS scales. J Pers Soc Psychol.

[33] Carver C.S. (2006). Measure of Current Status. http://www.psy.miami.edu/faculty/ccarver/sclMOCS.html.

[34] Cohen S., Kamarck T., Mermelstein R. (1983). A global measure of perceived stress. J Health Soc Behav.

[35] Tedeschi R.G., Calhoun L.G. (1996). The Posttraumatic Growth Inventory: measuring the positive legacy of trauma. J Trauma Stress.

[36] Feldman G., Hayes A., Kumar S., Greeson J., Laurenceau J.-P. (2007). Mindfulness and emotion regulation: the development and initial validation of the Cognitive and Affective Mindfulness Scale-Revised (CAMS-R). J Psychopathol Behav Assess.

[37] Harris P.A., Taylor R., Thielke R., Payne J., Gonzalez N., Conde J.G. (2009). Research electronic data capture (REDCap)--a metadata-driven methodology and workflow process for providing translational research informatics support. J Biomed Inform.

[38] Cherkin D, Sherman KJ, Balderson B (2016). Effect of mindfulness-based stress reduction vs cognitive behavioral therapy or usual care on back pain and functional limitations in adults with chronic low back pain: a randomized clinical trial. JAMA.

[39] Herman P.M., Anderson M.L., Sherman K.J., Balderson B.H., Turner J.A., Cherkin D.C. (2017). Cost-effectiveness of mindfulness-based stress reduction versus cognitive behavioral therapy or usual care among adults with chronic low back pain. Spine.

[40] Wolowacz S.E., Samuel M., Brennan V.K., Jasso-Mosqueda J.-G., Van Gelder I.C. (2011). The cost of illness of atrial fibrillation: a systematic review of the recent literature. Europace.

