# Supplementary material for: A SMART approach to reducing paroxysmal atrial fibrillation symptoms: Results from a pilot randomized controlled trial
Source: Heart Rhythm O2. 2021 Jun 22;2(4):326–32. doi: 10.1016/j.hroo.2021.06.003 (PMC8369288; doi:10.1016/j.hroo.2021.06.003)
Supplement: Supplemental Figure 1 [file mmc1.pdf]

Supplementary Figure 1: Study design and timepoints for study questionnaires/data collection: timepoint 1 (T1) at 0 months, timepoint 2 (T2) at 3 months, and timepoint 3 (T3) at 6 months. Both the immediate and waitlist control groups answered questionnaires (★) at T1 and T2. Due to funding limitations, only the waitlist control group answered questionnaires at T3.

Timepoint (months)	T1 (0)		T2 (3)		T3 (6)
Immediate Group	★	SMART Program	★		
Waitlist Control Group	★		★	SMART Program	★

★= when questionnaires were administered
